# METTL3/YTHDF2 m6A axis accelerates colorectal carcinogenesis through epigenetically suppressing YPEL5

**DOI:** 10.1002/1878-0261.12898

**Published:** 2021-01-25

**Authors:** Dan Zhou, Weiwei Tang, Yidan Xu, Yajie Xu, Binbin Xu, Shanshan Fu, Yanting Wang, Fangfang Chen, Yongxiong Chen, Yinshu Han, Gueyhorng Wang

**Affiliations:** ^1^ Research Center of Natural Cosmeceuticals Engineering Xiamen Medical College China; ^2^ Application Technique Engineering Center of Natural Cosmeceuticals, College of Fuijan Province Xiamen Medical College China; ^3^ Eye Institute of Xiamen University, Fujian Provincial Key Laboratory of Ophthalmology and Visual Science, Medical College Xiamen University China; ^4^ Department of Medical Oncology, Cancer Hospital, The First Affiliated Hospital of Xiamen University, School of Medicine Xiamen University, Teaching Hospital of Fujian Medical University China

**Keywords:** colorectal cancer, m6A modification, metastasis, prognostic biomarker

## Abstract

N6‐methyladenosine (m6A) has emerged as the most prevalent post‐transcriptional modification on mRNA that contributes prominently to tumorigenesis. However, the specific function of m6A methyltransferase methyltransferase‐like 3 (METTL3) in colorectal cancer (CRC) remains elusive. Herein, we explored the biological function of METTL3 in CRC progression. Clinically, METTL3 was frequently upregulated in CRC tissues, cell lines, and plasma samples and its high expression predicted poor prognosis of CRC patients. Functionally, knockdown of METTL3 significantly repressed CRC cell proliferation and migration *in vitro*, while its overexpression accelerated CRC tumor formation and metastasis both *in vitro* and *in vivo*. Mechanistically, METTL3 epigenetically repressed YPEL5 in an m6A‐YTHDF2‐dependent manner by targeting the m6A site in the coding sequence region of the YPEL5 transcript. Moreover, overexpression of YPEL5 significantly reduced CCNB1 and PCNA expression. Collectively, we identified the pivotal role of METTL3‐catalyzed m6A modification in CRC tumorigenesis, wherein it facilitates CRC tumor growth and metastasis through suppressing YPEL5 expression in an m6A‐YTHDF2‐dependent manner, suggesting a promising strategy for the diagnosis and therapy of CRC.

## Graphical abstract

Here, we demonstrate that methyltransferase‐like 3 (METTL3)‐catalyzed N6‐methyladenosine (m6A) modification facilitates tumor growth and metastasis in colorectal cancer (CRC). METTL3 can reduce YPEL5 expression in an m6A‐YTHDF2‐dependent manner. Our findings highlight a novel m6A regulatory mechanism, with ‘writer’ METTL3, ‘reader’ YTHDF2, and ‘target’ YPEL5 being promising diagnostic or therapeutic targets in CRC.

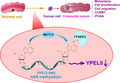

**Abbreviations**
CDS: coding sequences
COAD: colon adenocarcinoma
CRC: colorectal cancer
GEPIA: Gene Expression Profiling Interactive Analysis
H&E: hematoxylin–eosin
lgG: immunoglobulin G
m6A: N6‐methyladenosine
METTL14: methyltransferase‐like 14
METTL3: methyltransferase‐like 3
READ: rectum adenocarcinoma
RIP: RNA immunoprecipitation
siRNAs: small interfering RNAs
WTAP: Wilms tumor 1‐associated protein
YTHDF2: YTH domain family protein 2

## 1 Introduction

Colorectal cancer (CRC) is the third most commonly diagnosed cancer and the second leading cause of cancer death worldwide, with over 1.8 million new cases and 0.8 million deaths predicted in 2018 [1] . Although continuous advances in screening and treatment, the 5‐year survival rates are still low according to a later stage with distant metastasis at diagnosis [2]. Therefore, further studies are urgently needed to characterize the underlying molecular mechanism involved in CRC metastasis for the development of novel biomarkers and therapeutic approaches.

N6‐methyladenosine (m6A) has emerged as the most prevalent post‐transcriptional modification on mRNA and noncoding RNAs that affects RNA stability, splicing, and translational efficacy in eukaryotes [3, 4, 5, 6]. The m6A occurs mostly at the N6 position of adenosine in RRACH motif (R = G/A; H = A/C/U) [7, 8]. Moreover, the m6A is enriched around stop codons in 3′UTR, internal long exons, and precursor mRNAs, thereby participating in cell cycle regulation, cell differentiation, and cell fate decision [9, 10].

This modification is a reversible process on the basis of methyltransferases (writers), demethylases (erasers), and m6A binding proteins (readers) [11]. The core writers that catalyze the m6A modification are methyltransferase‐like 3 (METTL3), methyltransferase‐like 14 (METTL14), and Wilms tumor 1‐associated protein (WTAP) [12]. METTL3 acts as the catalytic subunit with S‐adenosylmethionine binding ability [13]. METTL14, a homolog of METTL3, contributes to stabilize METTL3 and bind RNA substrate [14]. WTAP is essential for localization of METTL3‐METTL14 heterodimer to nuclear speckle without enzymatic activity [15]. The modification can be reversed by demethylases (erasers) including fat mass and obesity‐associated protein and alkB homolog 5 [16, 17]. The YT521‐B homology (YTH) domain family proteins including YTHDF1, YTHDF2, YTHDF3, YTHDC1, and YTHDC2 are identified as m6A readers in the nucleus or cytoplasm [5, 18].

Recently, accumulating evidence has highlighted the potential roles of m6A regulators, especially METTL3, in CRC initiation, progression, and prognosis. METTL3 exhibited its cancer suppressive role in CRC through regulating p38/ERK pathways [19]. Interestingly, recent studies reported that METTL3 showed an oncogenic potentiality in promoting CRC tumorigenesis and progression [20, 21, 22]. Thus, the mechanism of METTL3 might be complicated and knowledge about the correlations between METTL3 and m6A readers is rather limited. Herein, by systematically investigating the biological function of METTL3 in CRC, we provide several novel insights into METTL3‐dominated m6A modification and the development of potential therapeutic strategies.

## 2 Materials and methods

### 2.1 Specimens and cell lines

A total of 109 primary CRC and 44 adjacent nontumorous tissue specimens obtained from patients undergoing tumor surgical resection at The First Affiliated Hospital of Xiamen University with histologically verified CRC between June 2013 and December 2017 were frozen immediately in liquid nitrogen and stored at −80 °C. Plasma specimens were collected before therapeutic intervention from an independent set of 20 CRC patients and 20 healthy subjects. All experimental procedures were approved by the Ethics Committee of School of Medicine in Xiamen University and followed the Declaration of Helsinki. Written informed consent was permitted by each participant after complete explanation of the study. Clinical information of all human participants was summarized in Table S1.

Human colonic epithelial cell line NCM460 and CRC cell lines HCT116, HT29, SW480, and SW620 were acquired from the Cancer Center of Xiamen University (Xiamen, China) and cultured in RPMI‐1640 medium (Hyclone, Beijing, China) supplemented with 10% FBS at 37 °C in a humidified atmosphere of 5% CO_2_. All five cell lines had been tested negative for mycoplasma contamination and authenticated by short tandem repeat fingerprinting.

### 2.2 Establishment of stable knockdown and overexpressed cell lines

Stable knockdown and overexpressed cell lines were obtained by lentiviral vector‐H1‐METTL3 short‐hairpin RNA‐GFP‐puro (LV3‐shMETTL3 1, LV3‐shMETTL3 2) and lentiviral vector‐EF1a‐METTL3‐GFP‐puro (LV5‐METTL3) delivery, respectively. Empty vectors (LV3‐NC, LV5‐NC) were applied as the negative controls. The shRNAs sequences were listed in Table S2. The overexpression vectors (pEnter‐YTHDF2, pEnter‐YPEL5) and the control vector were purchased commercially (Vigene Biosciences, Shangdong, China).

### 2.3 Transfection

Small interfering RNA (siRNA) targeting YTHDF2‐specific regions were reported previously and synthesized by Gene Pharma (Shanghai, China). Transfections were carried out using the Lipofectamine 2000 (Invitrogen, Carlsbad, CA, USA) following the manufacturer's instructions. The siRNA sequence was summarized in Table S2.

### 2.4 RNA extraction and quantitative PCR (qPCR)

Total RNA was isolated from cell lines, tissues, and plasma specimens by TRIzol reagent (Invitrogen). cDNA was synthesized using PrimeScript RT reagent Kit (TAKARA, Dalian, China). The relative target gene mRNA expression was analyzed using the comparative CT method, with GAPDH as the endogenous control. All the qPCR primer sequences were shown in Table S2.

### 2.5 Western blot

Proteins were lysed by cold RIPA buffer with a phosphatase inhibitor (Thermo Fisher Scientific, Waltham, MA, USA) and a proteinase inhibitor phenylmethanesulfonyl fluoride (Beyotime, Shanghai, China). The BCA protein assay was applied to measure protein concentrations (Thermo Scientific). The samples were separated by SDS/PAGE and transferred to Polyvinylidene fluoride membranes. The membranes were blocked in 5% BSA and incubated with the anti‐METTL3 (Abcam cat#:ab195352, Cambridge, UK), anti‐YPEL5 (Proteintech cat#:11730‐1‐AP, Wuhan, China), anti‐YTHDF2 (Proteintech cat#: 24744‐1‐AP), anti‐CCNB1 (Proteintech cat#:55004‐1‐AP), anti‐PCNA (Proteintech cat#:10205‐2‐AP), and anti‐GAPDH (Sigma cat#:G9295, St. Louis, MO, USA) overnight at 4 °C, followed by treatment of horseradish peroxidase‐conjugated secondary antibody (Sigma). The signal bands were detected with ECL kit (Lu long, China).

### 2.6 Tissue microarray (TMA) and Immunohistochemical analysis

The CRC TMA was employed as previously described, including 14 CRC tissues and matched normal tissues [23]. The TMA was stained with anti‐METTL3 (Abcam) and analyzed using a light microscope. The staining intensity was detected using the image‐pro plus 6.0 software (Media Cybernetics, Silver Spring, MD, USA). The relevant clinical information was provided in Table S3.

### 2.7 Wound healing assay

The transfected cells were seeded into 6‐well culture plates until 80% confluent. Scratches were carefully created through a sterile 200 μL tip. Photographs were taken at 0 and 24 h.

### 2.8 Transwell assay

Briefly, the transfected cells in 150 μL serum‐free medium were loaded in 24‐well transwell inserts with 8 μm diameter pores. Medium with 10% FBS was placed in the lower chamber. After 24 h, noninvasive cells were scraped and the invaded cells on the lower surface were stained with crystal violet solution and counted.

### 2.9 Colony formation assay

The transfected cells were resuspended at a density of 400 cells into each well of 6‐well plates. After 2 weeks, colonies were fixed and stained with crystal violet solution.

### 2.10 Cell proliferation assay

A total of 3000 transfected cells were cultured into 96‐well plates. After 24 h, the cells were added with counting kit‐8 solution (Dojindo, Kumamoto, Japan) following the manufacturer's instructions and incubated for additional 2 h. The absorbance values were measured at 450 nm using a spectrophotometer.

### 2.11 Cell cycle assay

The transfected cells were collected and fixed using ice‐cold 70% EtOH overnight at −20 °C. Then, the fixed cells were stained with propidium iodide at 4 °C for 30 min in the dark and assessed using flow cytometry (Beckman Coulter Inc., Brea CA, USA). The cell phase distribution was measured through modfit lt 3.1 software (Verity Software House, Inc., Topsham, ME, USA).

### 2.12 *In vivo* xenograft and metastasis model

For the xenograft model, METTL3 stable overexpressed SW620 cells (1 × 10^7^) or control cells were subcutaneously injected into the right axilla of the female anesthetized BALB/C nude mice (4–6 weeks old, 18–20 g, four mice per group), respectively. The body weight and tumor volumes (length × width^2^ × 0.5) were measured twice a week. After 21 days, all mice were sacrificed and tumors were surgically removed for hematoxylin–eosin (H&E) staining.

For the metastasis model, MTTL3 stable overexpressed SW620 cells (1 × 10^6^) or control cells were injected into the exposed spleen of the anesthetized BALB/C nude mice, respectively. After 21 days, liver metastases were carefully detected using a fluorescent stereoscope and embedded for H&E staining.

All animal experiments were approved by the Ethics Committee of Xiamen University Laboratory Animal Center.

### 2.13 RNA m6A modification quantification

RNA m6A methylation status in total RNA was detected using the m6A RNA methylation quantification kit (Abcam) in accordance with the manufacturer's instructions. Briefly, total RNA was bound to strip wells at 37 °C for 90 min. Then, a specific m6A capture antibody and detection antibody were added separately. The detected signal was enhanced by enhancer solution and then quantified by a microplate spectrophotometer at 450 nm.

### 2.14 M6A‐RNA Immunoprecipitation (MeRIP) qPCR Assay

RNA was chemically sheared into ~ 100nt size and was then incubated with m6A antibody‐coated magnetic beads or negative immunoglobulin G (lgG) following the standard protocol of Magna MeRIP™ m6A Kit (Merck Millipore cat#: 17‐10499, Billerica, MA, USA ). Enrichment of m6A containing mRNA was detected using qPCR. Primers targeting m6A enriched regions of YPEL5 were listed in Table S2.

### 2.15 RNA‐binding protein immunoprecipitation (RIP) assay

RIP assay was carried out using the EZ‐Magna RIP™ RNA‐Binding Protein Immunoprecipitation Kit according to the manufacturer's protocol (Merck Millipore cat#: 17‐700). In brief, cells (2 × 10^7^) were lysed and processed for immunoprecipitation overnight. The primers for qPCR were listed in Table S3.

### 2.16 Luciferase reporter assay

Stable METTL3 overexpressed SW620 and HT29 cell lines were cotransfected with plasmids containing coding sequences (CDS) of wide‐type or mutant (in which adenosine from m6A site was replaced by cytosine) YPEL5 fragments using Lipofectamine 2000 following the manufacturer's instructions. Luciferase activities were detected using the dual‐luciferase reporter assay kit (Beyotime) at 48 h after transfection and were normalized to Renilla fluorescence.

### 2.17 Bioinformatic analysis

Gene Expression Profiling Interactive Analysis (GEPIA) database was used to analyze METTL3 expression levels in pan‐cancer [24]. Using the box plots, a paired Student's *t*‐test was employed to assess the statistical difference in the METTL3 expression levels between tumor and matched the Cancer Genome Atlas normal samples. Correlation between METTL3 or YTHDF2 and YPEL5 was also calculated. A coefficient *R* > 0.3 or *R* < −0.3 represented a moderate correlation; and a coefficient *R* > 0.5 or *R* < −0.5 represented a large correlation. Protein–protein interactions among METTL3 and m6A writers were predicted using STRING database [25]. RNA m6A sites of YPEL5 were predicted using m6aVar and Gene Expression Omnibus (GEO) database [26, 27]. RNA‐binding proteins (RBPs) and their binding motifs were identified by Starbase database [28].

### 2.18 Statistical analysis

Data were reported as mean ± SD. Statistical analyses were carried out using graphpad software (GraphPad Software Inc., La Jolla, CA, USA). The two‐tailed Student's *t*‐test and one‐way ANOVA were used to calculate statistical significance. Mann–Whitney test was used to calculate the correlation between METTL3 expression and clinicopathologic characters. Pearson correlation was employed to analysis the correlation. Survival curves were plotted using the Kaplan–Meier method with the log‐rank test. *P* < 0.05 was considered statistically significant.

## 3 Results

### 3.1 Frequent upregulation of METTL3 in CRC

**Fig. 1:**
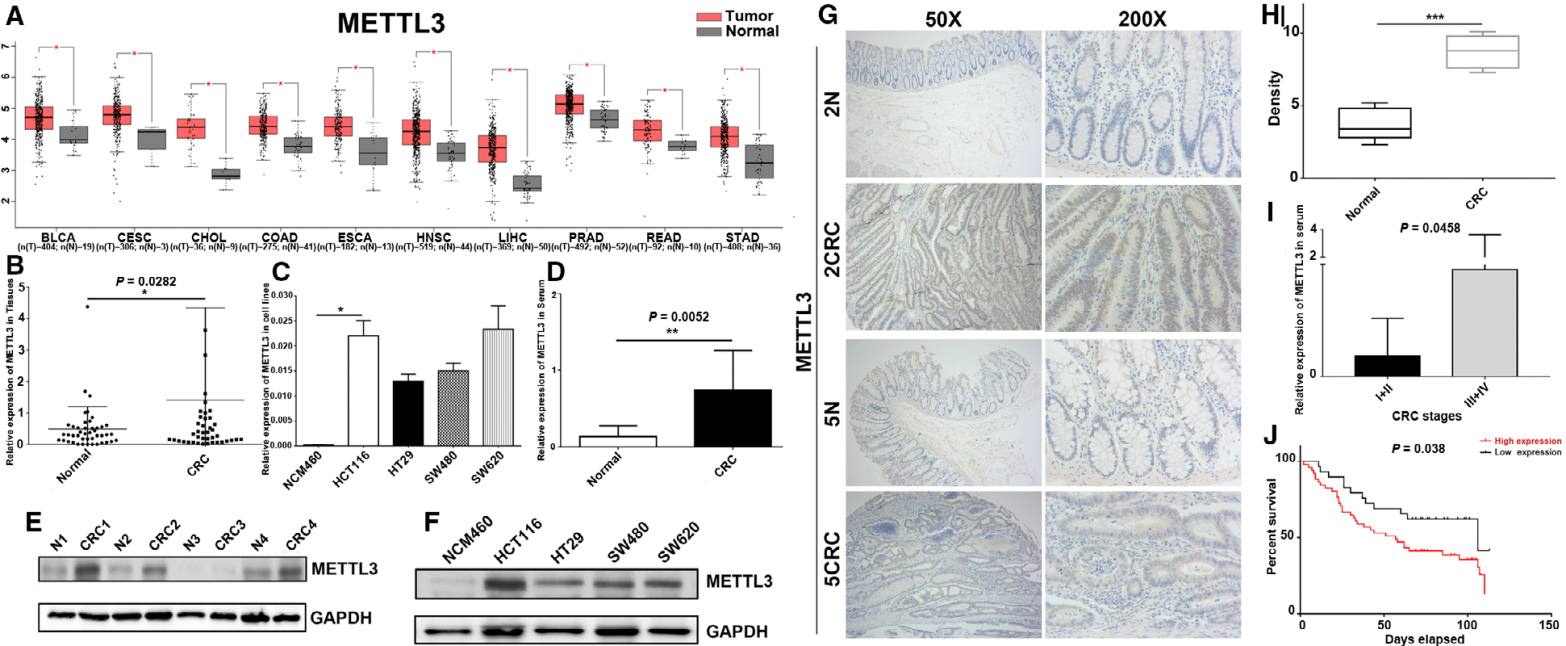
Frequent upregulation of METTL3 in CRC. (A) Overexpression of METTL3 was observed in 10 out of 31 cancer types. (B) METTL3 was markedly increased in 44 primary CRC tissues compared to paired normal controls. (C) Elevated METTL3 was detected in four CRC cell lines (HCT116, HT29, SW480, and SW620). (D) METTL3 upregulation was observed in cell‐free RNA from CRC plasma samples than that in healthy subjects. (E‐F) Representative western blot image for protein expression level of METTL3 in the same cohort of CRC tissues and cell lines. (G‐H) Immunohistochemistry staining of METTL3 in CRC tissues and paired normal tissues at 100 × and 400× magnification. (I) METTL3 mRNA expression was gradually increased from stage I to IV CRC. (J) Kaplan–Meier survival curve of overall survival in 80 CRC patients based on METTL3 expression analyzed by qPCR. Results were presented as means ± SD (*n* = 3 per group). The two‐tailed Student's *t*‐test and one‐way ANOVA were used to perform comparison between two groups and more groups, respectively. *P < 0.05, **P < 0.01; ***P < 0.001. [Colour figure can be viewed at wileyonlinelibrary.com]

In an attempt to elucidate the biological function of METTL3 involved in tumorigenesis, we obtained expression landscape of METTL3 across different cancer types with tumor and normal samples from GEPIA database. Notably, overexpression of METTL3 was observed in 10 out of 31 cancer types, including colon adenocarcinoma (COAD) and rectum adenocarcinoma (READ) (Fig. 1A and S1). Subsequently, we confirmed METTL3 upregulation in 44 pairs of primary CRC tissues and normal controls by qPCR. Consistent with the bioinformatics finding, the expression of METTL3 was markedly increased in primary CRC tissues (Fig. 1B). In addition, elevated METTL3 was detected in four CRC cell lines (HCT116, HT29, SW480 and SW620) relative to colonic NCM460 cells (Fig. 1C). Consistently, 50% (10/20) of patients with CRC had an obvious METTL3 upregulation in cell‐free RNA from CRC plasma samples than that in healthy subjects (Fig. 1D). In line with METTL3 mRNA expression patterns, elevation of the protein status of METTL3 was also observed in the same cohort of CRC tissues and cell lines (Fig. 1E,F). Moreover, METTL3 staining showed predominantly positive in the majority of CRC tissues with an average density of 8.743 ± 1.086 (Fig. 1G,H). Additionally, METTL3 mRNA expression was significantly increased in stage III and IV compared with stages I and II CRC (Fig. 1I). Furthermore, survival analysis showed that high METTL3 expression predicted poor overall survival in CRC (Fig. 1J). Interestingly, the expression level of METTL3 was negatively correlated with age (*P* = 0.0264) in CRC, while positively associated with M stage (*P* = 0.0009), lymph node metastasis (*P* = 0.0382; Table S4). With the above results, we concluded that METTL3 was frequently increased in CRC and might be implicated in CRC tumorigenesis and progression.

### 3.2 Knockdown of METTL3 suppressed CRC cell proliferation and migration

**Fig. 2:**
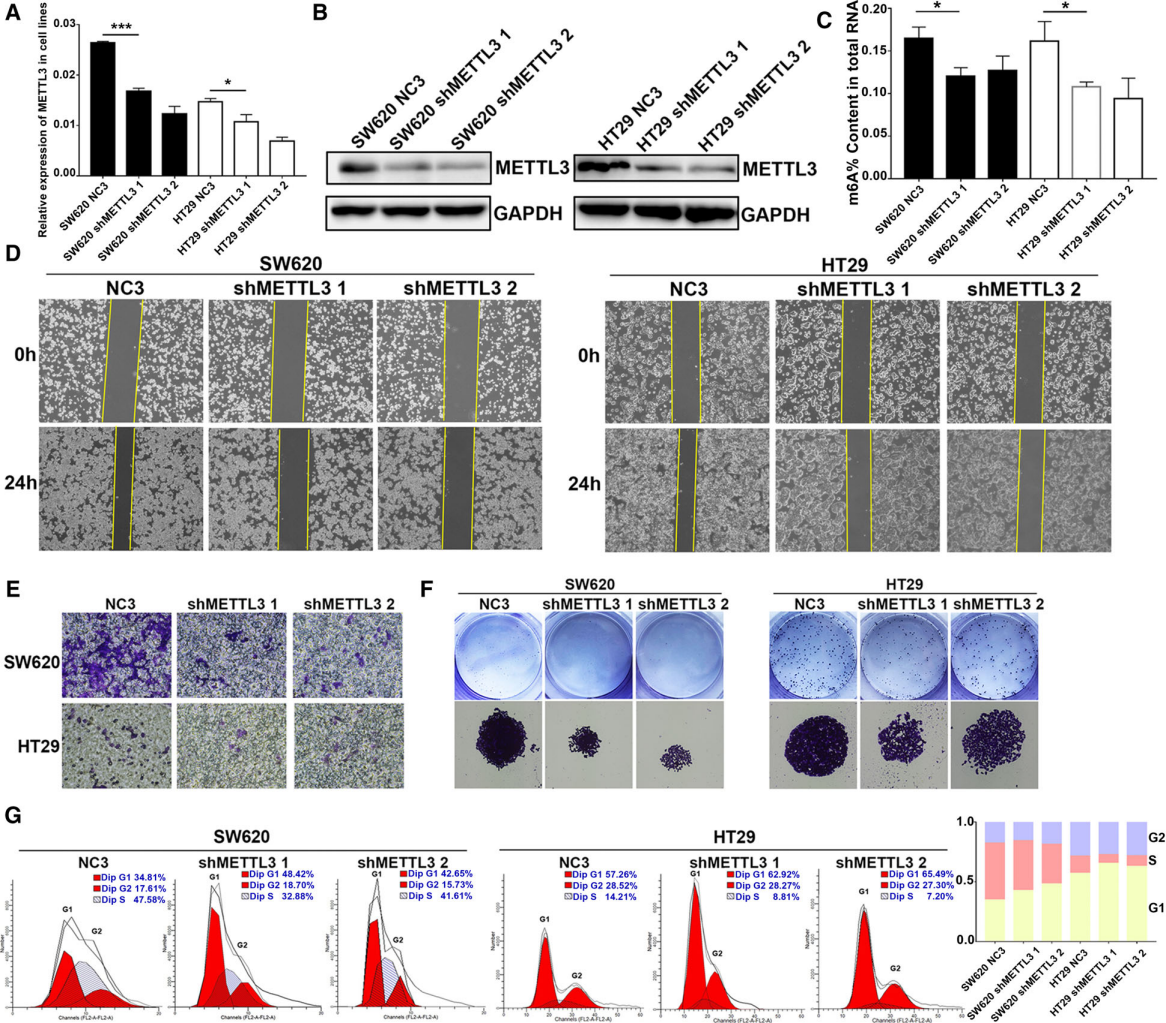
Knockdown of METTL3 suppressed CRC cell proliferation and migration. (A) The knockdown effect was verified at mRNA level using qPCR. (B) The knockdown effect was further verified at protein level using western blot. (C) Knockdown of METTL3 significantly decreased the mRNA m6A content. (D‐E) Wound healing assay and transwell assay showed the inhibition of migration in METTL3‐knockdown CRC cell lines. (F) Colony formation assay showed that METTL3 knockdown significantly repressed the cloning formation of CRC cells. (G) Cell cycle assay showed that knockdown of METTL3 increased the percentage of G1 of CRC cells. Results were presented as means ± SD (*n* = 3 per group). The two‐tailed Student's *t*‐test and one‐way ANOVA were used to perform comparison between two groups and more groups, respectively. *P < 0.05, ***P < 0.001. [Colour figure can be viewed at wileyonlinelibrary.com]

To explore the crucial function of METTL3 in CRC, we constructed stable METTL3 knockdown models in SW620 and HT29 cells using two distinct shRNA lentivirus (shMETTL3 1 and shMETTL3 2) and a control lentivirus (NC3). Successful knockdown of METTL3 was validated at both mRNA and protein levels (Fig. 2A,B). Correspondingly, knockdown of METTL3 significantly decreased the mRNA m6A content in SW620 and HT29 cells (Fig. 2C). Moreover, knockdown of METTL3 remarkably reduced cell migration and colony formation abilities (Fig. 2D–F). Similarly, knockdown of METTL3 increased the percentage of G1 and decreased the percentage of S‐G2 phase (Fig. 2G). Collectively, the above evidence suggested that METTL3 played a pivotal role in CRC cell proliferation and migration.

### 3.3 Overexpression of METTL3 facilitated CRC cell proliferation and migration

**Fig. 3:**
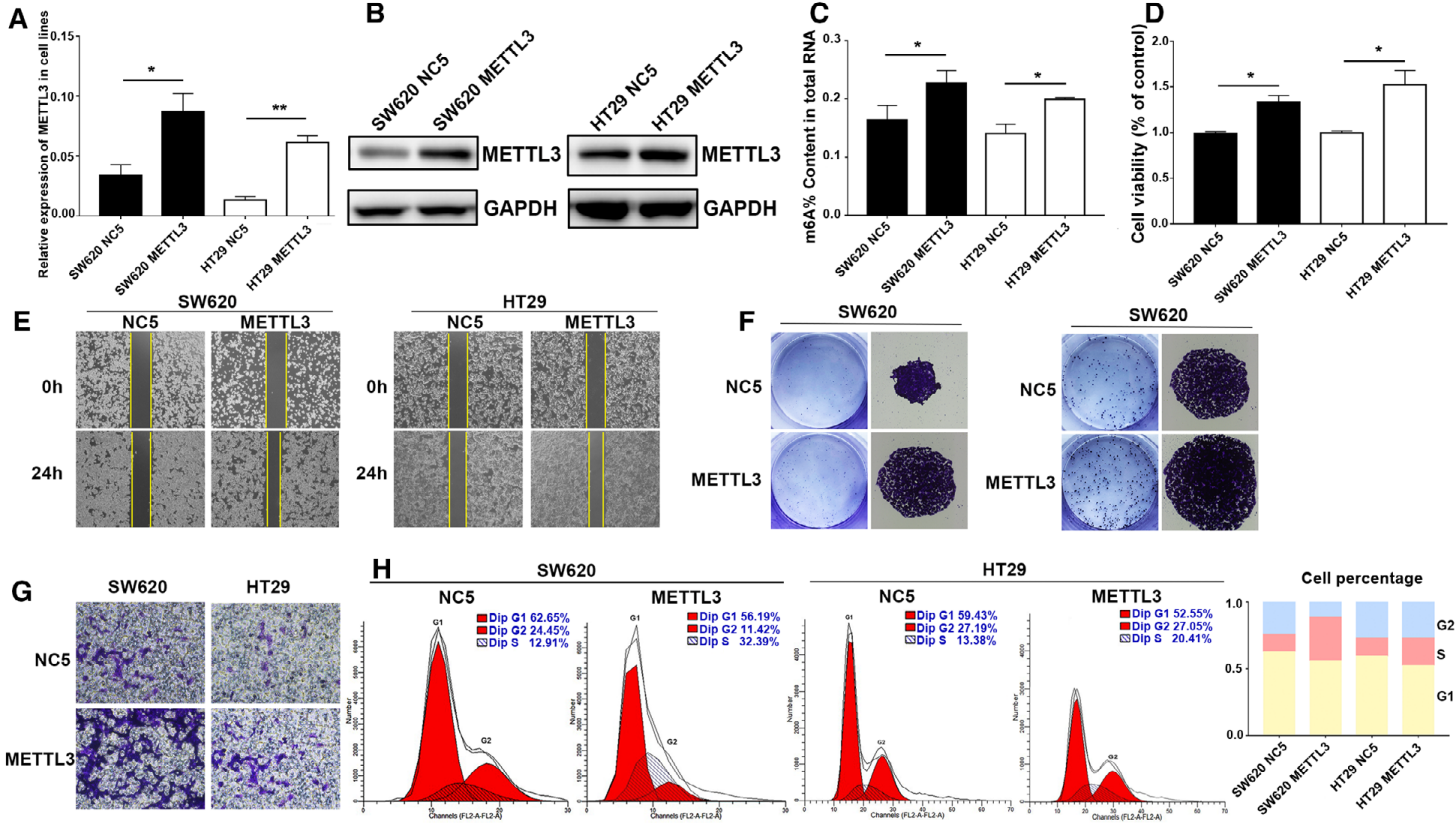
Overexpression of METTL3 facilitated CRC cell proliferation and migration. (A) The overexpression effect was verified at mRNA level using qPCR. (B) The overexpression effect was further verified at protein level using western blot. (C) Overexpression of METTL3 significantly increased the mRNA m6A content. (D) CCK8 assay showed that overexpression of METTL3 promoted CRC cell proliferation. (E) Wound healing assay showed the enhancement of migration in METTL3‐overexpressing CRC cell lines. (F) Colony formation assay showed that METTL3 overexpression significantly increased cloning formation of CRC cells. (G) Transwell assay further revealed that overexpression of METTL3 significantly promoted CRC cell migration. (H) Cell cycle assay showed that overexpression of METTL3 reduced the percentage of G1. Results were presented as means ± SD (*n* = 3 per group). The two‐tailed Student's *t*‐test and one‐way ANOVA were used to perform comparison between two groups and more groups, respectively. *P < 0.05, **P < 0.01. [Colour figure can be viewed at wileyonlinelibrary.com]

Subsequently, we established stable METTL3 overexpressing SW620 and HT29 cells. Overexpression of METTL3 was verified using qPCR and western blot (Fig. 3A,B). As expected, upregulation of METTL3 prominently elevated the mRNA m6A content (Fig. 3C). Moreover, overexpression of METTL3 markedly accelerated CRC cell proliferation, migration, and colony formation abilities (Fig. 3D‐G). In addition, overexpression of METTL3 reduced the percentage of G1 and increased the percentage of S‐G2 phase (Fig. 3H). All above findings indicated the oncogenic role of METTL3 in CRC cell growth and migration.

### 3.4 METTL3 promoted CRC tumor formation and metastasis *in vivo*

**Fig. 4:**
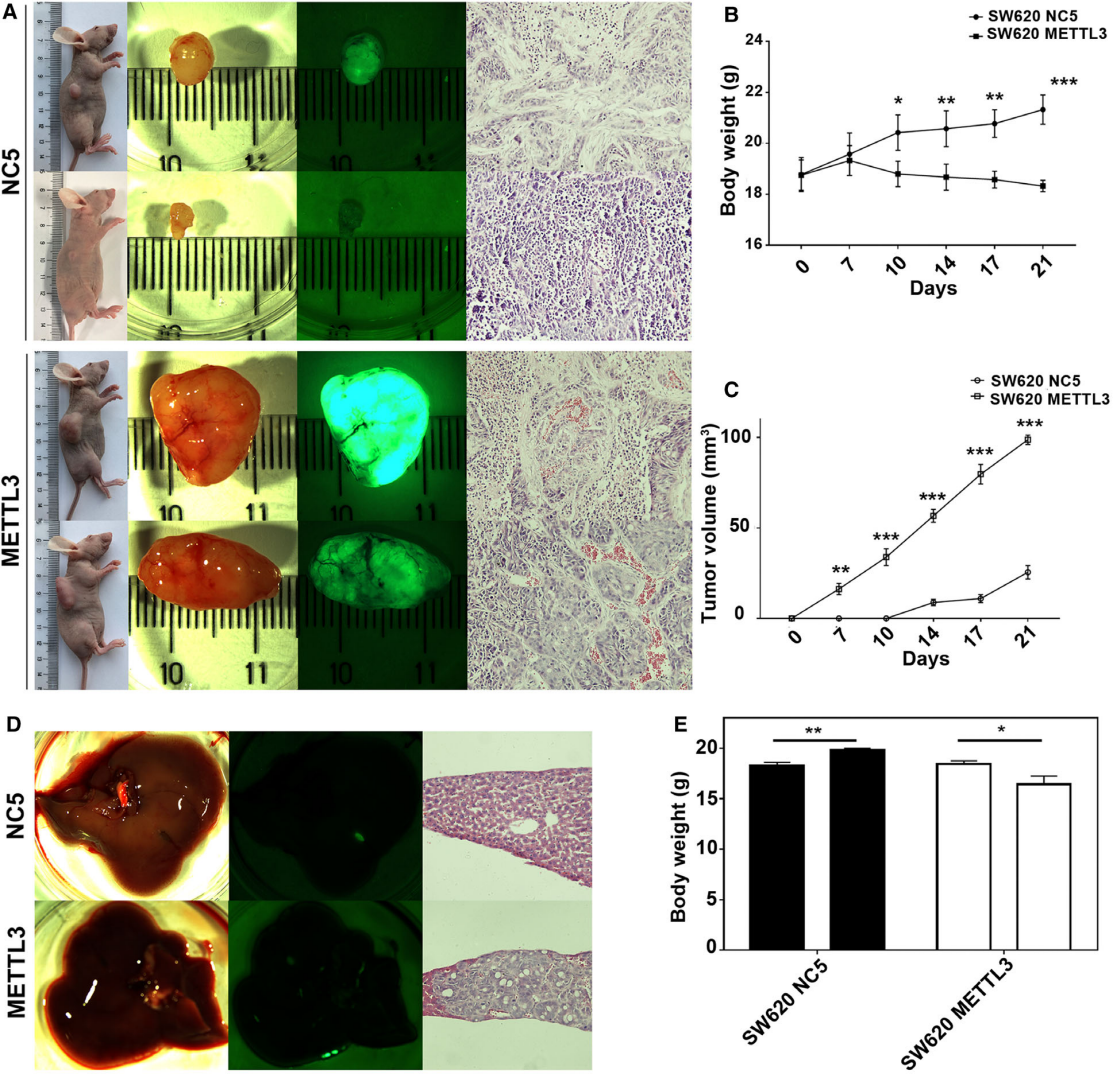
METTL3 promoted CRC tumor formation and metastasis *in vivo*. (A) Overexpression of METTL3 effectively increased CRC subcutaneous tumor formation and growth. (B) Overexpression of METTL3 significantly reduced the body weight of nude mice. (C) The tumor volume was dramatically increased on METTL3 overexpression. (D) Overexpression of METTL3 significantly promoted CRC liver metastasis. (E) The body weight loss of nude mice was obvious 21 days after METTL3‐overexpressing cells injection into the spleen. Results were presented as means ± SD (*n* = 4 per group). The two‐tailed Student's *t*‐test and one‐way ANOVA were used to perform comparison between two groups and more groups, respectively. *P < 0.05, **P < 0.01, ***P < 0.001. [Colour figure can be viewed at wileyonlinelibrary.com]

To further investigate the biological function *in vivo*, we performed a xenograft model and a metastasis model. The subcutaneous tumors injected with METTL3‐overexpressing cells developed more rapidly compared with those injected with control cells (Fig. 4A). The body weight of nude mice was significantly inhibited on METTL3 overexpression (Fig. 4B). Conversely, the tumor volume was remarkably increased (Fig. 4C). Moreover, overexpression of METTL3 notably promoted CRC liver metastasis compared to the control group (Fig. 4D). Similarly, the body weight loss of nude mice was obvious 21 days after METTL3‐overexpressing cells injection into the spleen (Fig. 4E). Thus, these results revealed that METTL3 was responsible for CRC tumor growth and metastatic *in vivo*.

### 3.5 YPEL5 was identified as a downstream target of METTL3

**Fig. 5:**
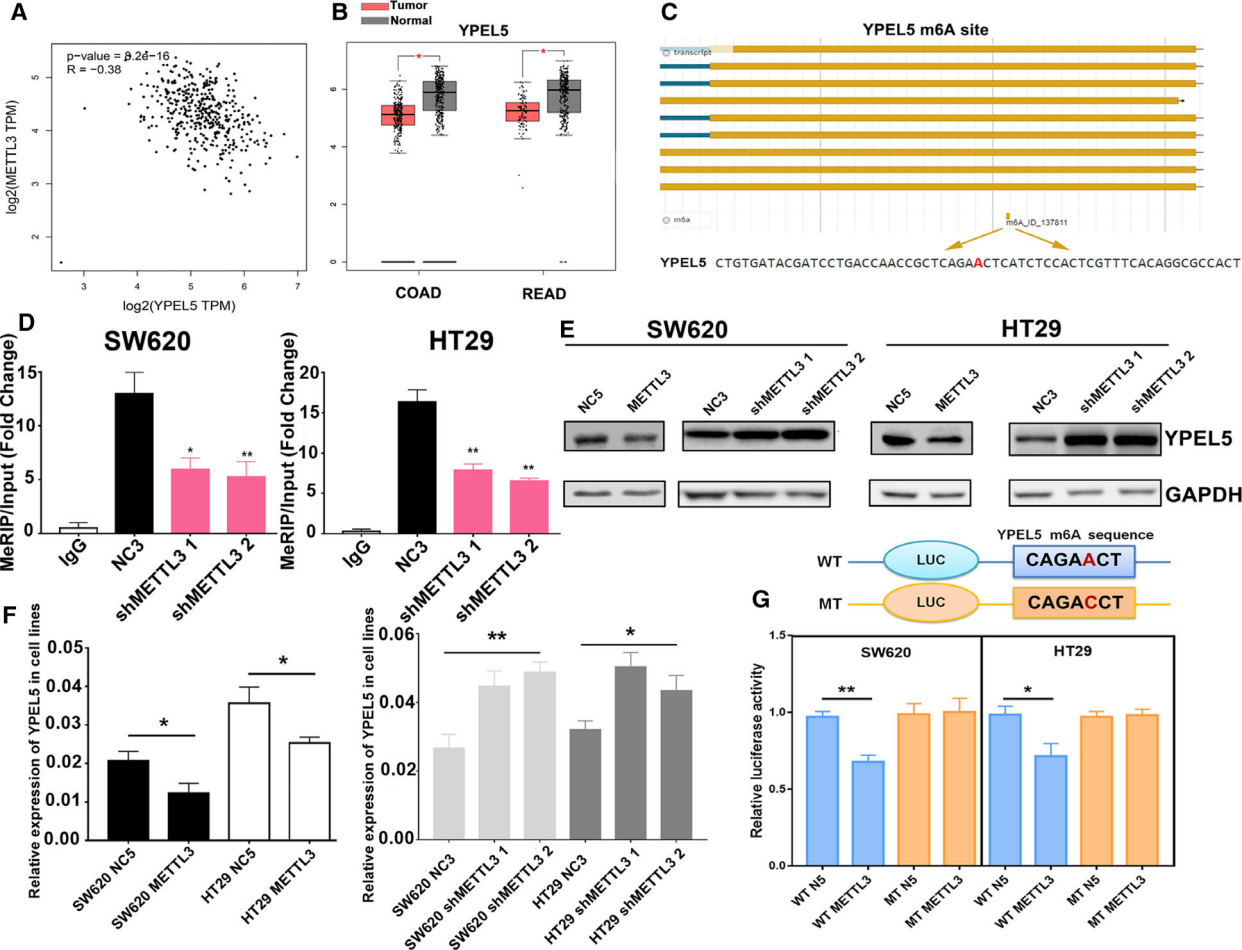
YPEL5 was identified as a downstream target of METTL3. (A) YPEL5 was shown to be significantly and negatively associated with METTL3 in GEPIA database. (B) The mRNA level of YPEL5 was dramatically decreased in COAD and READ as compared with normal controls. (C) An m6A motif site was found in the coding sequence region of YPEL5 through the m6aVar and RMBase V2.0 database. (D) MeRIP qPCR was used to examine the m6A level alterations of YPEL5 on METTL3 knockdown in SW620 and HT29. (E‐F) Overexpression of METTL3 prominently reduced both YPEL5 mRNA and protein expression, whereas knockdown of METTL3 increased YPEL5 expression, compared with normal controls. (G) Luciferase assay showed that overexpression of METTL3 repressed the expression of wide‐type YPEL5 reporter. Results were presented as means ± SD (*n* = 3 per group). The two‐tailed Student's *t*‐test and one‐way ANOVA were used to perform comparison between two groups and more groups, respectively. *P < 0.05, **P < 0.01. [Colour figure can be viewed at wileyonlinelibrary.com]

To elucidate the potential implication of METTL3 and identify the downstream target of METTL3 in CRC, we initially screen the correlation between METTL3 and candidate targets through GEPIA database. Thereinto, YPEL5 was predicted to be significantly and negatively related to METTL3 with the highest correlation coefficient (*R* = −0.38) (Fig. 5A). Moreover, the mRNA level of YPEL5 was dramatically decreased in COAD and READ as compared with normal controls (Fig. 5B). Then, integrating the results of the m6aVar and RMBase V2.0 database, we found an m6A motif site in the coding sequence region of YPEL5 (Fig. 5C). To confirm that METTL3 targets YPEL5 mRNA for m6A modification, MeRIP qPCR was carried out. The results suggested that the m6A‐specific antibody significantly enriched YPEL5 mRNA in contrast to the lgG control. Moreover, knockdown of METTL3 obviously decreased the m6A‐modified YPEL5 mRNA (Fig. 5D). In addition, we found that overexpression of METTL3 prominently reduced both YPEL5 mRNA and protein expression, whereas knockdown of METTL3 increased YPEL5 expression, compared with normal controls (Fig. 5E,F). Next, we established luciferase reporters containing the wide‐type and mutant YPEL5 to compare the effect of m6A modification on YPEL5 expression. The result showed that overexpression of METTL3 repressed the expression of wide‐type YPEL5 reporter, but failed to regulate the expression of the mutated YPEL5 reporter, indicating the regulation of YPEL5 expression level relied on METTL3‐associated m6A modification (Fig. 5G). Together, the accumulated findings suggested that METTL3 suppressed YPEL5 expression in an m6A‐dependent manner.

### 3.6 YTHDF2 preferentially decoded the m6A residue of YPEL5

**Fig. 6:**
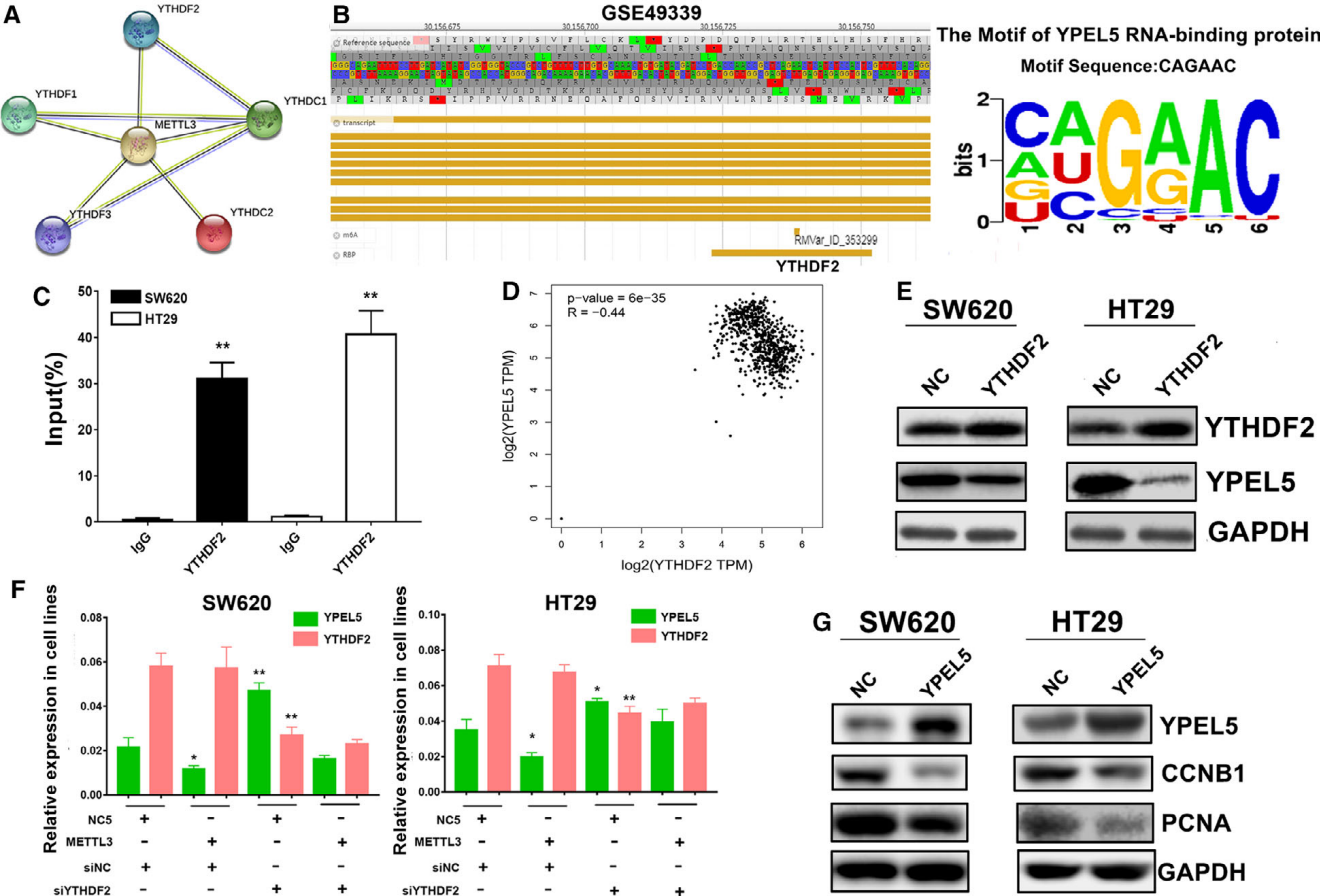
YTHDF2 preferentially decoded the m6A residue of YPEL5. (A) Protein–protein interaction network between METTL3 and m6A readers. (B) The YPEL5 m6A modification site was exactly in the YTHDF2 protein binding region. (C) RIP qPCR was conducted to confirm the YPEL5 mRNA enrichment by YTHDF2 in SW620 and HT 29 cells. (D) YTHDF2 expression was negatively associated with YPEL5 expression through bioinformatic analysis. (E) YPEL5 expression was significantly downregulated in YTHDF2‐overexpressing cells. (F) Relative YPEL5 and YTHDF2 mRNA levels were detected by qRT‐PCR in CRC cells transfected with lentiviruses carrying METTL3 and/or YTHDF2 siRNA (siYTHDF2). (G) Overexpression of YPEL5 strongly declined CCNB1 and PCNA expression. The two‐tailed Student's *t*‐test and one‐way ANOVA were used to perform comparison between two groups and more groups, respectively. Results were presented as means ± SD (*n* = 3 per group). *P < 0.05, **P < 0.01. [Colour figure can be viewed at wileyonlinelibrary.com]

The above results showed that alternations of METTL3 expression have been confirmed to result in significant changes of the mRNA m6A content. Previous study has reported that m6A status was dominated by the activities of m6A readers [29]. Protein–protein interaction network showed METTL3 could interact with five common m6A readers, including YTHDF1‐3 and YTHDC1‐2 (Fig. 6A). To investigate which m6A reader was selectively related to METTL3‐mediated m6A modification of YPEL5, we filtered the YPEL5 RBPs from GEO dataset GSE49339 in detail (Fig. 6B). Interestingly, the YPEL5 m6A modification site was exactly in YTHDF2 protein binding region. Furthermore, we performed a RIP qPCR assay to screen for YPEL5‐related m6A readers. The result validated that the YTHDF2 antibody specifically enriched the YPEL5 mRNA in SW620 and HT29 cells (Fig. 6C). Meanwhile, we found that YTHDF2 expression was negatively associated with YPEL5 expression (*R* = −0.44) (Fig. 6D). Furthermore, we analyzed the expression levels of YPEL5 in two stable YTHDF2‐overexpressing SW620 and HT29 cell lines. The results showed that YPEL5 expression was obviously downregulated in YTHDF2‐overexpressing cells (Fig. 6E). Next, we detected the mRNA expression levels of YPEL5 and YTHDF2 in METTL3 cells transfected with lentiviruses carrying METTL3 and/or YTHDF2 siRNA. The results showed that knockdown of YTHDF2 could significantly counteract the effects of elevated METTL3 on the expression of YPEL5 (Fig. 6F). Additionally, overexpression of YPEL5 strongly declined CCNB1 and PCNA expression (Fig. 6G). Collectively, these data revealed that METTL3‐mediated m6A modification suppressed YPEL5 expression through YTHDF2 in CRC.

## 4 Discussion

**Fig. 7:**
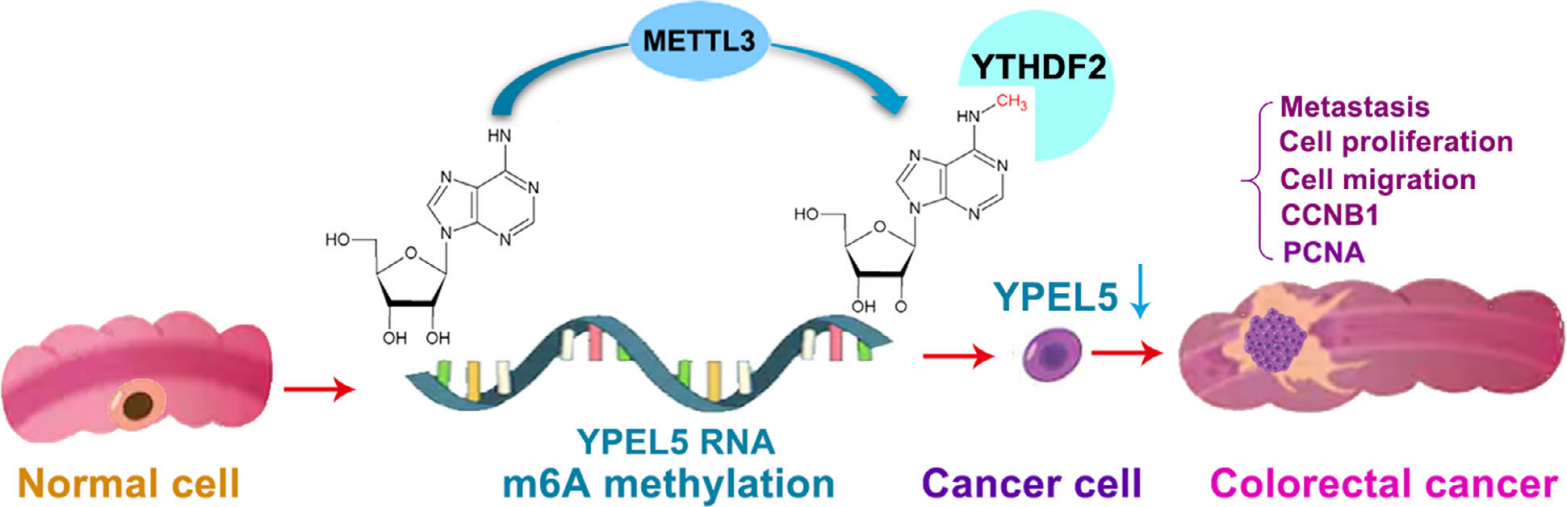
Working model of the proposed mechanism. [Colour figure can be viewed at wileyonlinelibrary.com]

RNA m6A methylation, the most prevailing RNA modification in mammals, participates in multiple aspects of RNA metabolism, containing RNA expression, splicing, translation, nuclear export, and decay [30, 31, 32]. Various m6A regulators, especially its methylase METTL3, have been highlighted and identified to be function either as oncogenes or tumor suppressors in diverse cancers. Owing to the different modification sites, various m6A binding readers and multiple downstream targets, complicated even contradictory results of m6A functions in carcinogenesis have been reported. For example, three independent studies showed that METTL3 acted as an oncogene in CRC, whereas METTL3 can also play a tumor suppressive role in CRC [19, 20, 21, 22]. In the present study, we revealed that METTL3 epigenetically repressed YPEL5 through m6A‐YTHDF2‐dependent manner. In line with previous findings of the oncogenic role of METTL3 in CRC, we unveiled that METTL3 was remarkably upregulated in CRC tissues, cell lines, and plasma samples and promoted CRC cell proliferation, migration, and metastasis thus contributing to CRC progression (Fig. 7). To the best of our knowledge, our paper is the first study on plasma levels of METTL3 mRNA in CRC patients.

In addition, we demonstrated that high expression of METTL3 predicted poor overall survival in CRC, suggesting that METTL3 was a candidate prognostic biomarker for CRC. Cell‐free mRNA circulated in plasma or serum samples has been identified to be noninvasive biomarkers for diagnosing and staging multiple cancers [33, 34]. Although the concentration of ribonucleases was high in blood, it was practical to detect cell‐free mRNA in plasma or serum of CRC patients due to the protection of microvesicles [35, 36]. Here, we for the first time found significantly increased levels of cell‐free METTL3 in the plasma from patients with CRC. The result further confirmed the potential value of METTL3 as noninvasive biomarkers in diagnosis of CRC.

In our bioinformatics analysis and luciferase assay confirmation, YPEL5 was assumed as the pivotal downstream target of METTL3. YPEL5 has been reported to be a member of highly conserved YPEL (Yippee‐like) gene family including five members that encoded zinc‐finger like metal binding proteins [37]. In cervical cancer cells, YPEL5 has been shown to play a suppressive role in cell proliferation and cell cycle progression [27]. Nevertheless, its exact function in CRC has never been elucidated. Importantly, our data revealed that overexpression of YPEL5 strongly declined CCNB1 and PCNA expression in SW620 and HT29 cells, indicating that YPEL5 was involved in the regulation of cell proliferation and cell cycle progression in CRC. However, further mechanism clarifying the significance of YPEL5 in regulation CRC progression deserves extensive study.

M6A readers have been demonstrated to recognize m6A sites, resulting in different destinies of mRNA [38]. As previous study reported, YTHDF2 induced the targeted degradation of the tumor suppressor to accelerate tumor progression, while this regulation was dependent on METTL3‐medicated m6A modification [5, 36, 37]. Through detailed bioinformatics analysis and further experiments confirmation, we found that METTL3‐dependent m6A modification suppressed the expression of YPEL5 through YTHDF2. Interestingly, our data also showed direct binding between YTHDF2 and the mRNA of YPEL5.

Although m6A sites preferentially appeared around near stop codons and in 3' UTR, a growing number of studies have demonstrated that m6A methylation in CDS may be more dynamic than those in 3′UTR [33]. Niu *et al*. [34] have reported that differentially methylated m6A is most often located in the CDS (54.10%) and followed by 5′UTR (21.71%). Mao *et al*. [35] have showed that genes participated in transcriptional regulation are overrepresented among mRNAs with CDS methylation. The importance of CDS is indubitable attribute to the elongation speed directly regulates the translational outcomes. In the present study, the m6A‐seq data from m6aVar and RMBase V2.0 database showed that an m6A motif site in the CDS of YPEL5 and overexpression of METTL3 failed to regulate the expression of the mutated YPEL5 reporter. However, it does not rule out the possibility that other m6A sites of YPEL5 can also be regulated by methyltransferase and recognized by different m6A readers. Thus, the roles of m6A in YPEL5 expression and other m6A regulators need further investigation.

## 5 Conclusions

Accordingly, we identified the indispensable role of METT3‐catalyzed m6A modification in CRC tumorigenesis, wherein it facilitates CRC tumor growth and metastasis. METTL3 could reduce YPEL5 expression in an m6A‐YTHDF2‐dependent manner. This m6A axis of ‘writer’ METTL3, ‘reader’ YTHDF2, and ‘target’ YPEL5 highlighted a novel m6A regulatory mechanism and could be a promising target for the diagnosis and therapy of CRC.

## Conflict of interest

The authors declare no conflict of interest.

## Author contributions

ZD performed molecular biology experiments and wrote the initial manuscript. TWW collected the plasma and tissue samples. XYJ performed additional experiments. XYD and XBB carried out related cell culture experiments. FSS and WYT participated in the statistical analysis. CFF conducted animal experiments. CYX participated in the design of this study. WGH and HYS helped to prepare the figures and revised the manuscript.

## Supporting information


**Fig. S1.** Expression landscape of METTL3 across the 20 rest cancer types between tumor and normal samples from GEPIA database.

**Table S1.** Clinical characteristics of patients with colorectal cancer and normal controls.

**Table S2**. shRNA, siRNA and primer sequences.

**Table S3**. Clinical characteristics of patients with colorectal cancer and normal controls in Tissue Array

**Table S4.** Association between clinicopathologic characteristics and METTL3 expression level.
