# Supplementary figures and images for: METTL3/YTHDF2 m6A axis accelerates colorectal carcinogenesis through epigenetically suppressing YPEL5
Source: Mol Oncol. 2021 Jan 25;15(8):2172–84. doi: 10.1002/1878-0261.12898 (PMC8333777; doi:10.1002/1878-0261.12898)

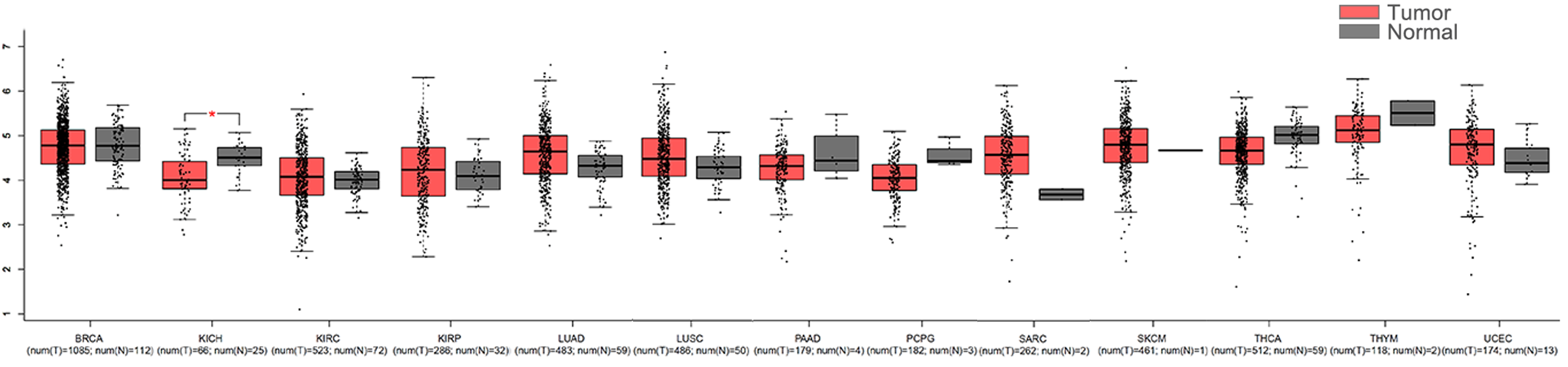

Supplement: Supplementary file 1 — Fig. S1. Expression landscape of METTL3 across the 20 rest cancer types between tumor and normal samples from GEPIA database. [file MOL2-15-2172-s003.tif]
